# Enhanced cytotoxicity of mitomycin C in human tumour cells with inducers of DT-diaphorase

**DOI:** 10.1038/sj.bjc.6690489

**Published:** 1999-06

**Authors:** X Wang, G P Doherty, M K Leith, T J Curphey, A Begleiter

**Affiliations:** Manitoba Institute of Cell Biology, Manitoba Cancer Treatment and Research Foundation and Departments of Pharmacology and Therapeutics and Internal Medicine, University of Manitoba, Winnipeg, MB, R3E 0V9, Canada; Department of Pathology, Dartmouth College, Hanover, NH, 03755, USA

**Keywords:** mitomycin C, DT-diaphorase, cytotoxicity, enzyme inducers

## Abstract

DT-diaphorase is a two-electron reducing enzyme that activates the bioreductive anti-tumour agent, mitomycin C (MMC). Cell lines having elevated levels of DT-diaphorase are generally more sensitive to MMC. We have shown that DT-diaphorase can be induced in human tumour cells by a number of compounds, including 1,2-dithiole-3-thione. In this study, we investigated whether induction of DT-diaphorase could enhance the cytotoxic activity of MMC in six human tumour cell lines representing four tumour types. DT-diaphorase was induced by many dietary inducers, including propyl gallate, dimethyl maleate, dimethyl fumarate and sulforaphane. The cytotoxicity of MMC was significantly increased in four tumour lines with the increase ranging from 1.4- to threefold. In contrast, MMC activity was not increased in SK-MEL-28 human melanoma cells and AGS human gastric cancer cells, cell lines that have high base levels of DT-diaphorase activity. Toxicity to normal human marrow cells was increased by 50% when MMC was combined with 1,2-dithiole-3-thione, but this increase was small in comparison with the threefold increase in cytotoxicity to tumour cells. This study demonstrates that induction of DT-diaphorase can increase the cytotoxic activity of MMC in human tumour cell lines, and suggests that it may be possible to use non-toxic inducers of DT-diaphorase to enhance the efficacy of bioreductive anti-tumour agents. © 1999 Cancer Research Campaign


*British Journal of Cancer* (1999) **80**(8), 1223–1230
© 1999 Cancer Research Campaign
Article no. bjoc.1999.0489

### Enhanced cytotoxicity of mitomycin C in human tumour cells with inducers of DT-diaphorase

**X Wang[1,2], GP Doherty[1,2], MK Leith[1,3], TJ Curphey[4] and A Begleiter[1,2,3]**

[1]Manitoba Institute of Cell Biology, Manitoba Cancer Treatment and Research Foundation and Departments of [2]Pharmacology and Therapeutics and [3]Internal Medicine, University of Manitoba, 100 Olivia Street, Winnipeg, MB, R3E 0V9 Canada; [4]Department of Pathology, Dartmouth College, Hanover, NH 03755 USA

**Summary** DT-diaphorase is a two-electron reducing enzyme that activates the bioreductive anti-tumour agent, mitomycin C (MMC). Cell lines having elevated levels of DT-diaphorase are generally more sensitive to MMC. We have shown that DT-diaphorase can be induced in human tumour cells by a number of compounds, including 1,2-dithiole-3-thione. In this study, we investigated whether induction of DT-diaphorase could enhance the cytotoxic activity of MMC in six human tumour cell lines representing four tumour types. DT-diaphorase was induced by many dietary inducers, including propyl gallate, dimethyl maleate, dimethyl fumarate and sulforaphane. The cytotoxicity of MMC was significantly increased in four tumour lines with the increase ranging from 1.4- to threefold. In contrast, MMC activity was not increased in SK-MEL-28 human melanoma cells and AGS human gastric cancer cells, cell lines that have high base levels of DT-diaphorase activity. Toxicity to normal human marrow cells was increased by 50% when MMC was combined with 1,2-dithiole-3-thione, but this increase was small in comparison with the threefold increase in cytotoxicity to tumour cells. This study demonstrates that induction of DT-diaphorase can increase the cytotoxic activity of MMC in human tumour cell lines, and suggests that it may be possible to use non-toxic inducers of DT-diaphorase to enhance the efficacy of bioreductive anti-tumour agents.

**Keywords:** mitomycin C; DT-diaphorase; cytotoxicity; enzyme inducers

Bioreductive anti-tumour agents are a new class of anticancer drugs, that have different chemical structures, but all require reductive activation by intracellular enzymes (Workman et al, 1993; Adams et al, 1994). These drugs are generally used to treat solid tumours, as they are often more toxic under hypoxic conditions (Workman et al, 1993). The prototype drug in this class is mitomycin C (MMC) (Rockwell et al, 1993), which is used in the treatment of breast (Hortobagyi, 1993), non-small-cell lung (Spain, 1993), head and neck (Coia, 1993), colorectal (Cummings et al, 1993) and gastric (Fujita et al, 1998) cancer. Interest in bioreductive agents has been raised by recent studies of combination treatment of MMC with radiation (Boyer, 1997), and by new agents, such as 3-hydroxymethyl-5-aziridinyl-1-methyl-2(1H-indole-4,7-dione)prop-β-en-α-ol (EO9) (Schellens, 1994) and tirapazamine (Bedikian et al, 1997; Miller et al, 1997). The one-electron reducing enzyme, NADPH:cytochrome P450 reductase (EC 1.6.2.4), may be the most important activating enzyme for MMC (Rockwell, 1993), but NAD(P)H:(quinone acceptor) oxidoreductase [EC 1.6.99.2] (DT-diaphorase), a two-electron reducing enzyme, is also a major activator in many systems (Begleiter et al, 1989, 1992; Riley and Workman, 1992; Ross et al, 1993). Other enzymes, such as NADH:cytochrome $b_5$ reductase [EC 1.6.2.2] (Hodnick, 1993) and xanthine dehydrogenase [EC 1.1.1.204] (Gustafson and Pritsos, 1992) may also play a role in activating MMC. Following activation, MMC can produce DNA cross-links (Tomasz et al, 1987; Ross, 1993) and DNA strand breaks (Lown et al, 1976; Pritsos et al, 1986); however, cross-linking appears to be the most important mechanism for anti-tumour activity (Rockwell et al, 1993).

*Received 4 September 1998*
*Revised 4 January 1999*
*Accepted 6 January 1999*

*Correspondence to:* A Begleiter, Manitoba Institute of Cell Biology, 100 Olivia Street, Winnipeg, Manitoba, Canada R3E 0V9

DT-diaphorase is a flavoprotein that catalyses two-electron reduction of quinones, quinone imines and nitrogen-oxides (Riley and Workman, 1992). It is also a phase II enzyme that detoxifies xenobiotics and carcinogens and protects cells from tumourigenesis (Beyer et al, 1988; Riley and Workman, 1992). Several diaphorases are known in humans, but NQO1 gene is the most extensively studied and seems to be the most important for activation of bioreductive agents (Jaiswal et al, 1990; Jaiswal, 1991; Belinsky and Jaiswal, 1993). Tumour cells generally have higher enzyme activity than the corresponding normal cells, but enzyme levels in primary tumours are low compared to levels in some tumour cell lines (Belinsky and Jaiswal, 1993; Ross et al, 1994; Smitskamp-Wilms et al, 1995). DT-diaphorase can be induced in many tissues by a wide variety of chemicals, including 1,2-dithiole-3-thione (D3T) (Egner et al, 1994), quinones, isothiocyanates, diphenols and Michael reaction acceptors (Prestera et al, 1993), many of which are dietary components. Much attention has been focused on the use of inducers of DT-diaphorase and other phase II detoxifying enzymes in cancer prevention. Oltipraz, a D3T analogue, has been shown to induce phase II detoxifying enzymes and is currently undergoing clinical trials as a chemopreventive agent (Kensler and Helzlsouer, 1995). Isothiocyanates like sulforaphane, which is found in broccoli, can also induce phase II enzymes (Prestera et al, 1993; Manson et al, 1997), and have been shown to prevent the formation of carcinogen-induced tumours in animals (Zhang et al, 1994).

DT-diaphorase plays an important role in activating bioreductive anti-tumour agents like MMC (Begleiter et al, 1989; Ross et al, 1993; Mikami et al, 1996; Nishiyama et al, 1997), EO9 (Plumb

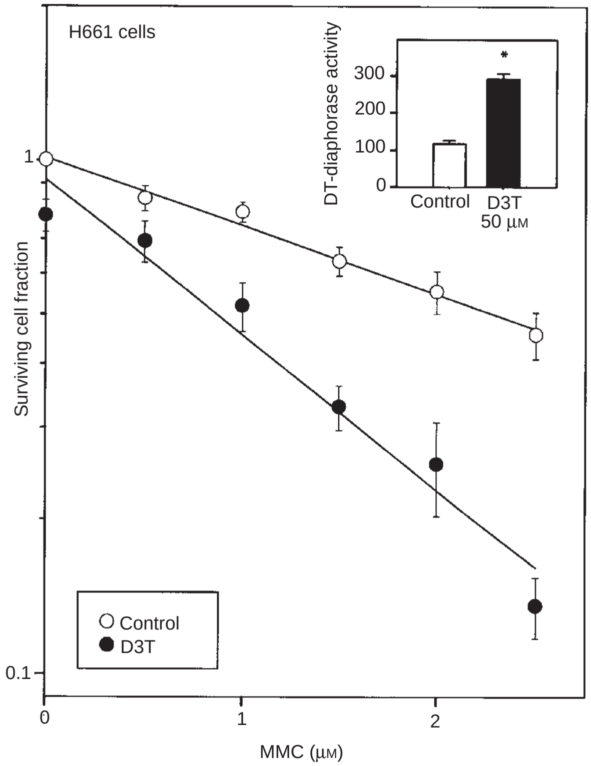

**Figure 1** Combination therapy with D3T and MMC in H661, human non-small-cell lung cancer cells. Cells were incubated at 37°C with, or without, 50 µM D3T for 48 h, then were treated with various concentrations of MMC for 1 h. Surviving cell fraction was determined by MTT assay. DT-diaphorase activity was measured as described in Materials and Methods and is shown in the inserted Figure. Data represent the mean ± s.e.m. of five determinations. The lines are linear regression lines. Two-tailed *t*-tests were used to determine the significance of the difference of the DT-diaphorase activities. (*$P < 0.05$)

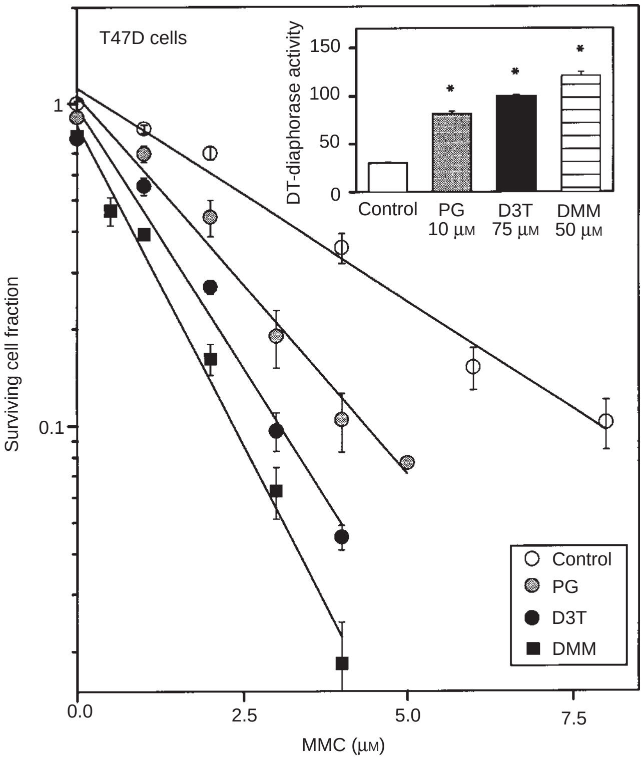

**Figure 2** Combination therapy with PG, D3T, or DMM, and MMC in T47D, human breast cancer cells. Cells were incubated with, or without, 10 µM PG, 75 µM D3T or 50 µM DMM for 48 h at 37°C, then were treated with various concentrations of MMC for 1 h. Surviving cell fraction was determined by MTT assay. DT-diaphorase activity was measured as described in Materials and Methods and is shown in the inserted Figure. Data represent the mean ± s.e.m. of three to eight determinations. The lines are linear regression lines. ANOVA analysis was used to determine the significance of the difference of the DT-diaphorase activities. (*$P < 0.05$)

et al, 1994), streptonigrin (Beall et al, 1996) and RH1 (Winski et al, 1998). Cell lines with high levels of DT-diaphorase are more sensitive to MMC (Begleiter et al, 1989; Ross et al, 1993; Mikami et al, 1996), and studies have shown a good correlation between the level of DT-diaphorase activity and the sensitivity to MMC in human tumour cell lines (Fitzsimmons et al, 1996). Transfecting the NQO1 gene into Chinese hamster ovary cells (Belcourt et al, 1996) and gastric carcinoma (Mikami et al, 1996) increased MMC cytotoxic activity to the cells.

Previously, we showed that D3T selectively increased the activity of DT-diaphorase in L5178Y murine lymphoma cells compared to mouse marrow cells. Pretreating these cells with D3T resulted in a twofold increase in MMC and a sevenfold increase in EO9 cytotoxicity with no effect on marrow toxicity (Begleiter et al, 1996). We also found that D3T induced DT-diaphorase activity in 28 of 38 human tumour cell lines representing ten tissue types (Doherty et al, 1998). Induction of DT-diaphorase activity in human tumour cells by D3T significantly increased the cytotoxicity of EO9 in these cells with no increase in toxicity to normal kidney cells (Doherty et al, 1998).

These findings indicate that inducers of DT-diaphorase could be used to enhance the anti-tumour efficacy of bioreductive agents. In this study, we investigated combination treatment of MMC and D3T in variety of human tumour cell lines, and studied dietary and pharmaceutical inducers of DT-diaphorase in human tumour cells. Although these inducers have been extensively studied in chemoprevention, they have not been investigated in combination with bioreductive agents.

### MATERIALS AND METHODS

### Materials

Cell culture media and fetal bovine serum (FBS) were obtained from GibcoBRL (Grand Island, NY, USA). All reagents for the DT-diaphorase assay, 3-[4,5-dimethythiazol-2yl]-2,5-diphenyltetrazolium bromide (MTT), 13-*cis*-retinoic acid, genistein, ursolic acid, ibuprofen, caffeic acid, folic acid and MMC were from Sigma (St Louis, MO, USA). Sulforaphane was purchased from LKT Labs, Inc. (St Paul, MN, USA). Protein concentration was measured using the Bio-Rad DC Kit (Bio-Rad, Mississauga, ON, Canada) with γ-globulin as standard. Vitamin K, dimethyl maleate (DMM), dimethyl fumarate (DMF), propyl gallate (PG), chalcone and aspirin were purchased from Aldrich (St Louis, MO, USA). MethoCult GF H4434 was obtained from Stem Cell Technologies Inc (Vancouver, BC, Canada). MMC was dissolved in phosphate-buffered saline (PBS): dimethyl sulphoxide (DMSO) (1:1, v/v). The final concentration of DMSO did not exceed 1%.

 © 1999 Cancer Research Campaign

### Cell culture

NCI-H661, non-small-cell lung carcinoma cells were obtained from American Type Culture Collection (Rockville, MD, USA) and were grown in RPMI-1640 and 10% FBS. HCT116, colon carcinoma cells and SK-MEL-28, malignant melanoma cells, were from American Type Culture Collection and were grown in DMEM/F12 (1:1) and 10% FBS. AGS, gastric adenocarcinoma cells, were obtained from Dr JA Wright (Manitoba Institute of Cell Biology, Winnipeg, MB, Canada) and were grown in RPMI-1640 and 10% FBS. T47D, breast ductal carcinoma cells, were from Dr S Mai (Manitoba Institute of Cell Biology) and were grown in RPMI-1640 and 10% FBS. HS578T, breast ductal carcinoma cells, were obtained from Dr S Pan (Greenebaum Cancer Center, Baltimore, MD, USA) and were grown in DMEM/F12 (1:1) and 10% FBS. Normal human bone marrow was obtained from marrow donated for transplantation and marrow cells were isolated by Ficoll–Hypaque separation (Begleiter et al, 1995). Marrow cells were cultured in RPMI-1640 and 10% FBS.

### Induction of DT-diaphorase

Cells were incubated with, or without, DT-diaphorase inducers at 37°C in 5% carbon dioxide for 48 h. The concentrations of inducers used were not toxic to the cells during the incubation time. Following incubation, cells were washed with PBS, suspended in 200 μl of 0.25 M sucrose, sonicated and stored at –80°C. Protein concentration was measured using the Bio-Rad DC Kit with γ-globulin as standard, then DT-diaphorase activity was measured spectrophotometrically by a modification of the procedure of Prochaska and Santamaria (Doherty et al, 1998; Prochaska et al, 1988) using menadione as the electron acceptor. DT-diaphorase activity was reported as dicoumarol-inhibitable activity and expressed as nmol $min^{-1}$ mg $protein^{-1}$. A dicoumarol concentration of 10 μM was used.

### Enzyme assays

T47D cells were incubated with, or without, 50 μM DMM for 48 h. Glutathione S-transferase (GST) activity was measured in supernatant from cell sonicates by a spectrophotometric procedure using 1-chloro-2,4-dinitrobenzene as substrate (Habig et al, 1974). NADPH:cytochrome P450 reductase activity was measured in supernatant from cell sonicates by a spectrophotometric assay using cytochrome c as the electron donor (Strobel and Digman, 1978). NADH:cytochrome $b_5$ reductase activity was determined by a previously described spectrophotometric procedure (Barham et al, 1996). Xanthine dehydrogenase activity was measured using a spectrophotometric method, in which xanthine dehydrogenase

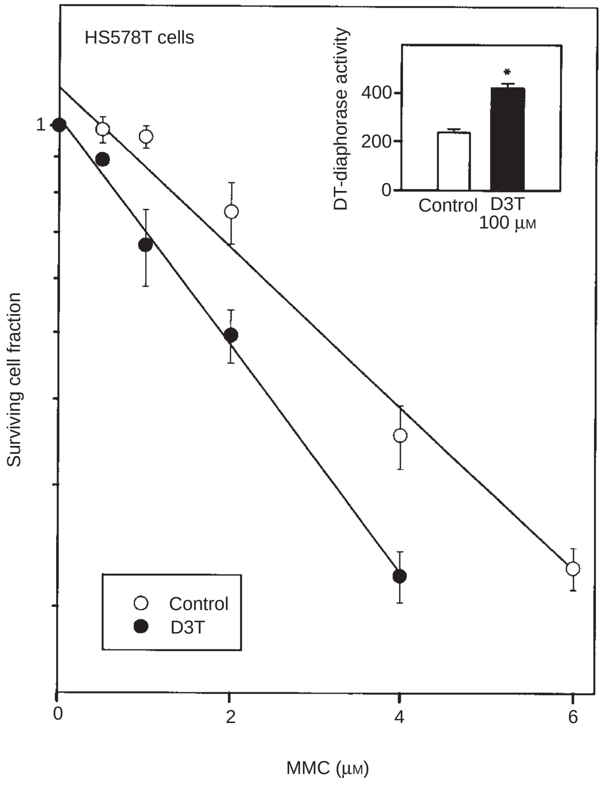

**Figure 3** Combination therapy with D3T and MMC in HS578T, human breast cancer cells. Cells were incubated at 37°C with, or without, 100 μM D3T for 48 h, then were treated with various concentrations of MMC for 1 h. Surviving cell fraction was determined by MTT assay. DT-diaphorase activity was measured as described in Materials and Methods and is shown in the inserted Figure. Data represent the mean ± s.e.m. of three to six determinations. The lines are linear regression lines. Two-tailed *t*-tests were used to determine the significance of the difference of the DT-diaphorase activities. (*$P < 0.05$)

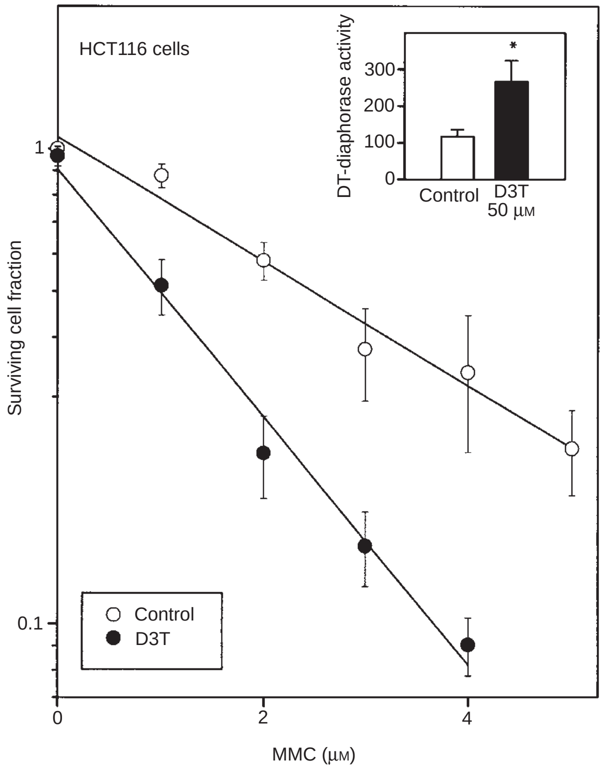

**Figure 4** Combination therapy with D3T and MMC in HCT116, human colon cancer cells. Cells were incubated at 37°C with, or without, 50 μM D3T for 48 h, then were treated with various concentrations of MMC for 1 h. Surviving cell fraction was determined by MTT assay. DT-diaphorase activity was measured as described in Materials and Methods and is shown in the inserted Figure. Data represent the mean ± s.e.m. of four to six determinations. The lines are linear regression lines. Two-tailed *t*-tests were used to determine the significance of the difference of the DT-diaphorase activities. (*$P < 0.05$)

*© 1999 Cancer Research Campaign*

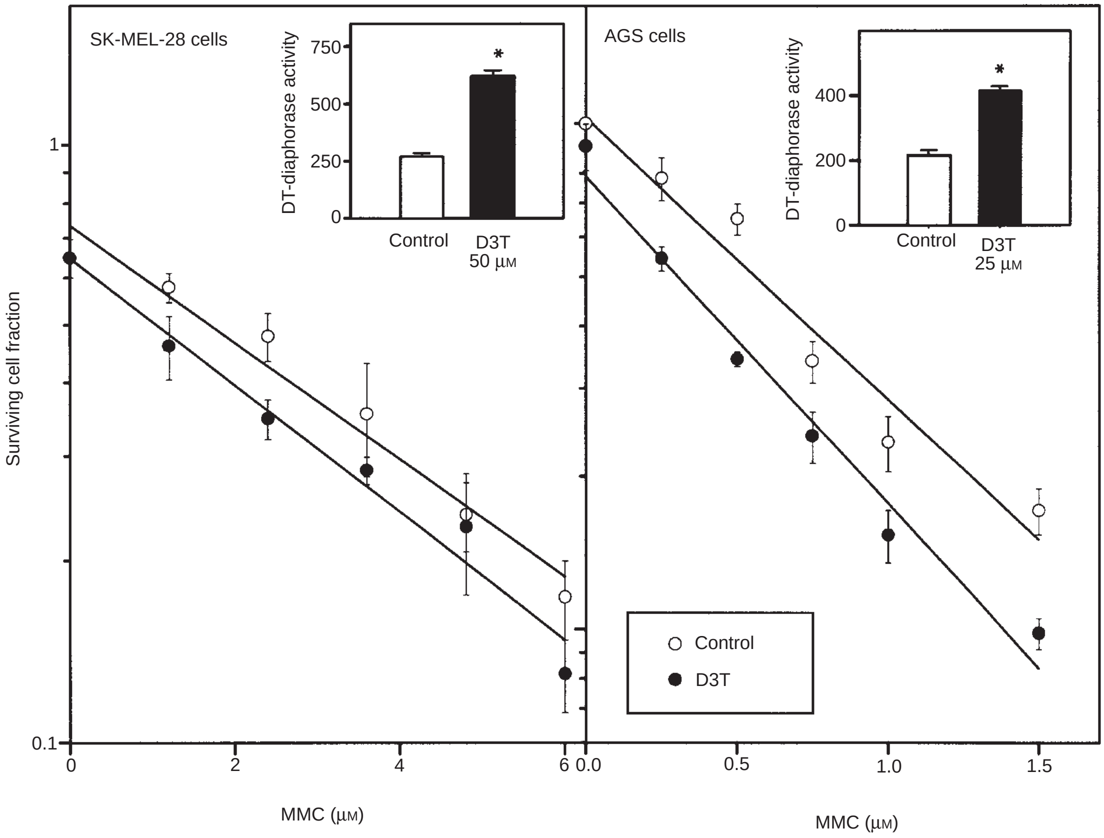

**Figure 5** Combination therapy with D3T and MMC in SK-MEL-28, human melanoma cells and AGS, human stomach cancer cells. SK-MEL-28 cells were incubated at 37°C with, or without, 50 μM D3T for 48 h, then were treated with various concentrations of MMC for 1 h. AGS, human stomach cancer cells were incubated at 37°C with, or without, 25 μM D3T for 48 h, then were treated with various concentrations of MMC for 1 h. Surviving cell fraction was determined by MTT assay. DT-diaphorase activities were measured as described in Materials and Methods and are shown in the inserted Figures. Data represent the mean ± s.e.m. of four determinations. The lines are linear regression lines. Two-tailed *t*-tests were used to determine the significance of the difference of the DT-diaphorase activities in control and D3T treated cells. (**P* <0.05)

and xanthine oxidase forms of the enzyme were distinguished by the formation of uric acid from xanthine in the presence and absence of $NAD^+$ (Gustafson et al, 1992).

### Cytotoxicity studies

Tumour cells were incubated with, or without, inducers for 4 [illegible] and then were treated with various concentrations of MMC for [illegible] at 3[illegible]°C. The surviving cell fraction was determined by MTT assay (Kirkpatrick et al, 1990; Johnston et al, 1994) after 4–9 days; this length of time was sufficient to allow at least three cell doublings. Normal human marrow cells were treated with D3T for 4[illegible] then incubated with various concentrations of MMC for [illegible] surviving cell fraction was determined by methylcellulose clonogenic assay (Begleiter et al, 1995). The $D_0$ (concentration of drug required to reduce the surviving cell fraction to 0.37) was calculated from the linear regression line of the surviving cell fraction versus drug concentration curve.

### RESULTS

### Combination treatment with D3T and MMC

H661, human non-small-cell lung cancer cells, were incubated with, or without, [illegible] μM D3T and then were treated with MMC. DT-diaphorase activity was increased from [illegible] ± 8.8 to [illegible]292.[illegible] ± 14[illegible] nmol $min^{-1}$ mg $protein^{-1}$ ($P < 0.001$), and the cytotoxicity of MMC was increased by 2.3-fold (Figure 1). The $D_0$ was 3.[illegible] ± 0.19 μM without D3T and 1.4[illegible] ± 0.15 μM with D3T ($P < 0.001$) (Table 1).

T47D, human breast cancer cells, were incubated with, or without, 75 μM D3T and then were treated with MMC. DT-diaphorase activity was increased from 30.[illegible] ± 0.8 to 10[illegible] ± 1.5 nmol $min^{-1}$ mg $protein^{-1}$ ($P < 0.001$), and the cytotoxicity of MMC was increased by 2.4-fold (Figure 2). The $D_0$ was 3[illegible] ± 0.[illegible] μM without D3T and 1.3[illegible] ± 0.[illegible] μM with D3T ($P < 0.001$) (Table 1).

HS578[illegible], human breast cancer cells, were incubated with, or without, [illegible]0 μM D3T and then were treated with MMC. DT-diaphorase activity was increased from 237.[illegible] ± 13.9 to [illegible] ± 19.0 nmol $min^{-1}$ mg $protein^{-1}$ ($P < 0.001$), and the cytotoxicity of MMC was increased by 40% (Figure 3). The $D_0$ was 3[illegible] ± 0.30 μM without D3T and 2.63 ± 0.[illegible] μM with D3T ($P < 0.01$) (Table 1).

HCT 116, human colon cancer cells, were incubated with, or without, [illegible] μM D3T and then were treated with MMC. DT-diaphorase activity was increased from [illegible] ± 17.9 to [illegible]267.[illegible] ± 56.3 nmol $min^{-1}$ mg $protein^{-1}$ ($P < 0.05$), and the cytotoxicity of MMC was increased by twofold (Figure 4). The $D_0$ was 3.3[illegible] ± 0.25 μM without D3T and 1.67 ± 0.[illegible] μM with D3T ($P < 0.001$) (Table 1).

SK-MEL-28, human melanoma, were incubated with, or without, [illegible] μM D3T and then were treated with MMC. DT-diaphorase

 *© 1999 Cancer Research Campaign*

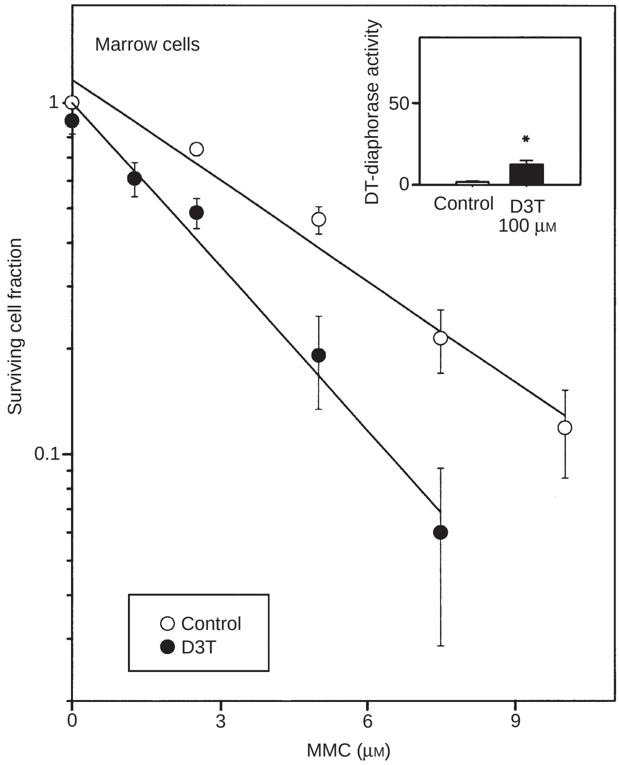

**Figure 6** Combination therapy with MMC and D3T in normal human marrow cells. Cells were incubated at 37°C with, or without, 100 μM D3T for 48 h, then were treated with various concentrations of MMC for 1 h. Surviving cell fraction was measured by methylcellulose clonogenic assay. DT-diaphorase activity was measured as described in Materials and Methods and is shown in the inserted figure. Data represent the mean ± s.e.m. of four to five determinations. The lines are linear regression lines. Two-tailed *t*-tests were used to determine the significance of the difference of the DT-diaphorase activities. (*$P < 0.05$)

activity was increased from 270.0 ± 14.7 to 622.2 ± 22.9 nmol min$^{-1}$ mg protein$^{-1}$ ($P < 0.001$). AGS, human gastric cancer cells, were incubated with, or without, 25 μM D3T and then were treated with MMC. DT-diaphorase activity was increased from 216.5 ± 14.4 to 413.8 ± 13.4 nmol min$^{-1}$ mg protein$^{-1}$ ($P < 0.001$). However, there was no significant increase in MMC cytotoxicity in either cell line (Figure 5 and Table 1).

### Induction of DT-diaphorase by dietary inducers in T47D human tumour cells

The ability of dietary components and pharmaceuticals to induce DT-diaphorase was examined in T47D human breast cancer cells. The base level of DT-diaphorase in this cell line was 27.8 ± 1.2 nmol min$^{-1}$ mg protein$^{-1}$, and eight of 14 inducers showed significant induction of enzyme activity (Table 2). The induced enzyme levels ranged from 40.8 ± 1.2 to 128.5 ± 5.6 nmol min$^{-1}$ mg protein$^{-1}$. The best inducers were DMM and DMF, with induced enzyme levels of 121.0 ± 3.9 and 128.5 ± 5.6 nmol min$^{-1}$ mg protein$^{-1}$ respectively.

### Combination treatment of MMC with dietary inducers

T47D cells were incubated with, or without, 10 μM PG or 50 μM DMM and then were treated with MMC. DT-diaphorase activity increased from 30.0 ± 0.8 to 81.1 ± 2.8 and 121.0 ± 3.9 nmol min$^{-1}$ mg protein$^{-1}$ ($P < 0.001$), and the cytotoxicity of MMC was increased by two- and threefold for PG and DMM respectively (Figure 2). The $D_0$ was 3.27 ± 0.20 μM with MMC alone, 1.85 ± 0.14 μM with PG and 1.13 ± 0.09 μM with DMM ($P < 0.001$) (Table 1).

### Effect of DMM on NADPH:cytochrome P450 reductase, GST, NADH:cytochrome $b_5$ reductase and xanthine dehydrogenase activity

Incubation with 50 μM DMM at 37°C for 48 h had no significant effect on the levels of NADPH:cytochrome P450 reductase and NADH:cytochrome $b_5$ reductase activity in T47D cells. The activity of xanthine dehydrogenase was too low to be detected in this cell line. There was a small increase in GST activity after incubation with DMM with GST levels increasing from 20.2 ± 0.6 to 24.0 ± 1.1 nmol min$^{-1}$ mg protein$^{-1}$ ($P < 0.05$).

### Bone marrow toxicity

Normal human marrow cells were incubated with 100 μM D3T and then were treated MMC. DT-diaphorase activity increased from 1.9 ± 0.3 to 12.7 ± 2.4 nmol min$^{-1}$ mg protein$^{-1}$ ($P < 0.005$) (Figure 6). The surviving cell fraction of human marrow progenitor cells (CFU-G, CFU-M, CFU-GM, CFU-E and BFU-E) was determined by methylcellulose clonogenic assay. The $D_0$ was 4.66 ± 0.41 μM without D3T and 2.95 ± 0.23 μM with D3T ($P < 0.05$) (Figure 6 and Table 1). There was no obvious selective effect on any of the progenitor cell types.

### DISCUSSION

DT-diaphorase is a highly inducible enzyme that plays an important role in activation of bioreductive anti-tumour drugs. It is also a phase II detoxifying enzyme, which prevents carcinogenesis by detoxifying reactive carcinogens. Elevated levels of DT-diaphorase activity have been shown to increase the cytotoxicity of MMC (Begleiter et al, 1989; Ross et al, 1993). We have previously shown that pretreatment with D3T, an inducer of DT-diaphorase, significantly increased the cytotoxic activity of E09 in mouse (Begleiter et al, 1996) and human tumour cells (Doherty et al, 1998), with little or no effect on normal mouse marrow (Begleiter et al, 1996) or normal human kidney cells (Doherty et al, 1998).

In this study, we extended these investigations to examine combination therapy with MMC and D3T in six human tumour cell lines. D3T increased DT-diaphorase activity in all six cell lines, and pretreatment with the enzyme inducer significantly enhanced the cytotoxicity of MMC in four of the cell lines. Combination treatment with D3T and MMC increased the cytotoxic activity of MMC by 2.3-fold in H661 non-small-cell lung cancer cells, by 2.4-fold in T47D breast cancer cells, by 1.4-fold in HS578T breast cancer cells and by twofold in HCT116 human colon cancer cells. These results demonstrate that this combination treatment is effective in enhancing the cytotoxicity of MMC in different tumour types. D3T did not increase MMC cytotoxic activity in SK-MEL-28 melanoma cells or AGS stomach cancer cells. Both these cell lines have relatively high base levels of DT-diaphorase activity. This suggests that this combination therapy approach may be restricted by the level of DT-diaphorase in the cells. If the base or induced level of DT-diaphorase is above an upper threshold, further induction may not lead to an increase in

© 1999 Cancer Research Campaign

**Table 1** Effect of inducers of DT-diaphorase on the cytotoxicity of MMC in human cells

| | | | $D_0$ (µM) | | |
|---|---|---|---|---|---|
| **Tumour type** | **Cell line** | **Inducer** | **Control** | **With inducer** | ***P*-value** |
| Colon | HCT116 | D3T | 3.31 ± 0.25 | 1.67 ± 0.11 | < 0.001 |
| Lung | H661 | D3T | 3.24 ± 0.19 | 1.43 ± 0.15 | < 0.001 |
| Skin | SK-MEL-28 | D3T | 4.40 ± 0.41 | 4.06 ± 0.34 | NS |
| Stomach | AGS | D3T | 0.78 ± 0.08 | 0.66 ± 0.05 | NS |
| Breast | HS578T | D3T | 3.72 ± 0.30 | 2.63 ± 0.10 | < 0.01 |
| Breast | T47D | D3T | 3.27 ± 0.20 | 1.34 ± 0.13 | < 0.001 |
| Breast | T47D | DMM | 3.27 ± 0.20 | 1.13 ± 0.09 | < 0.001 |
| Breast | T47D | PG | 3.27 ± 0.20 | 1.85 ± 0.14 | < 0.001 |
| Normal human bone marrow | | D3T | 4.66 ± 0.41 | 2.95 ± 0.23 | < 0.05 |

Cells were treated with, or without, inducers at 37°C for 48 h, then were incubated with various concentrations of MMC for 1 h. Surviving cell fractions were measured by MTT assay for tumour cells or by methylcellulose clonogenic assay for normal marrow cells. The cytotoxic activity of MMC is presented as the $D_0$, which was obtained from the linear regression line of the surviving cell fraction vs drug concentration curve. The data represent the mean ± s.e.m. of three to eight determinations. A *t*-test comparing the significance of the difference of the slopes of the linear regression lines was used to compare the $D_0$ for cells treated with, or without, inducers. NS, not significant.

**Table 2** Induction of DT-diaphorase in T47D human breast cancer cells by dietary inducers

| **Inducers** | **Concentration** | **Enzyme activity (nmol min$^{-1}$ mg protein$^{-1}$)** | ***P*-value** |
|---|---|---|---|
| Control | | 27.8± 1.2 | |
| D3T | 100 µM | 101.0± 2.8 | <0.001 |
| 13-*cis*-retinoic acid | 10 µM | 61.6 ± 4.9 | <0.001 |
| Genistein | 5 µM | 23.8 ± 1.4 | NS |
| Ursolic acid | 15 µM | 29.6 ± 4.4 | NS |
| Chalcone | 20 µM | 41.4 ± 3.4 | <0.001 |
| Aspirin | 1 mM | 40.8 ± 1.2 | <0.001 |
| Ibuprofen | 200 µM | 16.1 ± 1.1 | NS |
| Caffeic acid | 500 µM | 33.4 ± 2.3 | NS |
| Folic acid | 100 µM | 17.3 ± 2.5 | NS |
| Vitamin K | 10 µM | 17.3 ± 1.3 | NS |
| Dimethyl maleate (DMM) | 50 µM | 121.0 ± 3.9 | <0.001 |
| Propyl gallate (PG) | 10 µM | 80.5 ± 5.3 | <0.001 |
| Dimethyl fumarate (DMF) | 50 µM | 128.5 ± 5.6 | <0.001 |
| Sulforaphane | 10 µM | 102.3 ± 0.7 | <0.001 |

Cells were incubated with, or without, inducers at the concentrations shown at 37°C for 48 h. Cells were washed, pelleted, suspended in 200 µl of 0.25 M sucrose and sonicated. DT-diaphorase activity was measured using menadione as the electron acceptor. Data represent the mean ± s.e.m. of three to five determinations. Two-tailed *t*-tests were used to compare the significance of the difference of DT-diaphorase activity in control and inducer-treated cells. NS not significant.

MMC cytotoxicity. Our results, and a previous study (Beall et al, 1995), suggest that this upper threshold may be close to 300 nmol min$^{-1}$ mg protein$^{-1}$. While this may limit the use of this combination therapy approach in some situations, it should not significantly impair the use of DT-diaphorase inducers to increase the anti-tumour activity of bioreductive agents in the clinic since primary tumours generally have base levels of DT-diaphorase that are <100 nmol min$^{-1}$ mg protein$^{-1}$ (Schlager and Powis, 1990; Malkinson et al, 1992; Ross et al, 1994; Smitskamp-Wilms et al, 1995; Marin et al, 1997).

To further improve the clinical potential of this treatment strategy for increasing the activity of bioreductive agents, we investigated dietary components and some pharmaceuticals as non-toxic inducers of DT-diaphorase. Fourteen compounds were tested for their ability to induce DT-diaphorase in T47D cells, a cell line that has been shown to be readily inducible and has a relatively low base level of DT-diaphorase. Eight of the 14 compounds significantly increased DT-diaphorase activity with some of the compounds proving to be better inducers than D3T. DMM and DMF were the best inducers studied and produced fivefold increases in enzyme activity. These compounds are metabolites of fumaric and maleic acid that are commonly found in foods. Sulforaphane, which is extracted from broccoli and has been extensively studied as a potent chemopreventive agent, showed good enzyme induction, and PG, an antioxidant added to foods, produced a 2.5-fold increase in enzyme activity. Although some vitamins have been shown to increase DT-diaphorase activity (Wang and Higuchi, 1995), only 13-*cis*-retanoic acid was able to induce DT-diaphorase in T47D cells. There has been great interest in non-steroidal anti-inflammatory drugs (NSAIDs) in cancer prevention, but the mechanisms through which these agents work is still not clear. Thus, we tested aspirin and ibuprofen in this study to see if they could increase DT-diaphorase activity in tumour cells. Aspirin produced a small but significant induction of DT-diaphorase, but ibuprofen had no effect. Soybeans

 *© 1999 Cancer Research Campaign*

have been shown to increase phase II enzymes, including DT-diaphorase, in rats (Appelt and Reicks, 1997); however, genistein, which is extracted from soybeans, did not increase DT-diaphorase activity in our study. Manson et a (1997) have shown that caffeic acid is a moderate inducer of phase II enzymes, but in our study caffeic acid did not increase DT-diaphorase activity.

To investigate if the level of enhancement of MMC activity is dependent on the level of DT-diaphorase induction, we carried out combination therapy studies with PG, D3T and DMM together with MMC in T47D cells. Pretreatment of cells with these enzyme inducers increased MMC activity in the order PG < D3T < DMM, and this paralleled the increase in DT-diaphorase activity. This suggests that there is a relationship between the level of induction in enzyme activity and the enhancement of MMC cytotoxicity, provided the DT-diaphorase activity does not exceed an upper threshold level. Since primary tumours usually have lower levels of DT-diaphorase activity than tumour cells grown in vitro, it may be possible to achieve greater enhancement of anti-tumour activity of bioreductive agents in the clinic by using more potent inducers of DT-diaphorase.

Since many enzymes can activate bioreductive anti-tumour agents, we examined the effect of DMM on enzymes that have been reported to be involved in MMC activation. When T47D cells were treated with 50 μM DMM, there were no changes in NADPH:cytochrome P450 reductase, or NADH:cytochrome $b_5$ reductase activity. Xanthine dehydrogenase activity was too low to be detected either before or after DMM treatment. GST is a phase II enzyme that can be induced coordinately with DT-diaphorase. This enzyme can also protect cells from toxins by removing them from the cell, and has been shown to play a role in resistance to a number of anti-tumour agents, including MMC (Xu et al, 1994). Although we did not see an increase in GST activity in HL-60 human leukaemia cells following treatment with 100 μM D3T (Doherty et al, 1998), in this study there was an increase in GST activity when T47D cells were treated with DMM. However, the effect was small and there was still a large enhancement of MMC cytotoxic activity in these cells.

The major toxicity associated with the use of MMC is bone marrow toxicity. We have previously shown that D3T did not increase the toxicity of MMC to mouse bone marrow (Begleiter et al, 1996). In this study, pretreatment with D3T produced a 1.5-fold increase in toxicity to human marrow cells; however, this was small compared with the enhancement of MMC cytotoxic activity in T47D, H661 and HCT116 cells. This result suggests that combination therapy with DT-diaphorase inducers and MMC may increase the therapeutic index for MMC for appropriate tumours. In addition, DT-diaphorase does not appear to be the major enzyme involved in activating MMC. Thus, we would expect a greater increase in therapeutic index if this approach were used with bioreductive agents that are selectively activated by DT-diaphorase like E09 (Doherty et al, 1998) or RH1 (Winski et al, 1998). Indeed, we did see a greater enhancement of EO9 cytotoxic activity compared with MMC in mouse lymphoma cells pretreated with D3T (Begleiter et al, 1996).

These studies support the hypothesis that inducers of DT-diaphorase could be used to increase the effectiveness of bioreductive agents in the clinic; however, a number of concerns must still be addressed. Activation of bioreductive agents by one-electron reducing enzymes like NADPH:cytochrome P450 reductase is increased in the absence of oxygen because redox cycling of the reduced intermediates is prevented. We have shown that DT-diaphorase does not contribute to the activation of MMC under hypoxic conditions (Begleiter et al, 1992), and there is some evidence that this enzyme may actually decrease the activity of bioreductive agents under these conditions (Plumb et al, 1994). Since bioreductive agents have often been used to target hypoxic cells in solid tumours, induction of DT-diaphorase might result in decreased anti-tumour activity. However, only a small proportion of tumour cells in solid tumours are actually anoxic, with most cells being exposed to at least some levels of oxygen. Marshall and Rauth (1986) demonstrated that oxygen levels of <1% were sufficient to allow redox cycling and reverse the enhanced MMC cytotoxic activity under hypoxic conditions. Thus, increasing the level of DT-diaphorase is likely to increase the overall anti-tumour effectiveness of MMC. In addition, the activity of bioreductive agents that were specifically activated by DT-diaphorase would be unaffected by the level of oxygen in the tumour cells and would be increased by induction of DT-diaphorase.

Our studies have demonstrated that induction of DT-diaphorase can increase the cytotoxic activity of bioreductive agents in many tumours in vitro (Begleiter et al, 1996; Doherty et al, 1998). However, Nishiyama et al (1993) found a negative correlation between DT-diaphorase activity and MMC anti-tumour activity in vivo. In contrast, Malkinson et al (1992) saw greater MMC activity in human tumour xenografts with higher DT-diaphorase activity. Thus, the ability of DT-diaphorase inducers to enhance the anti-tumour activity of bioreductive agents must still be demonstrated in vivo. In addition, our findings suggest that this strategy may not be effective with all tumours, and that for clinical application it will be necessary to measure the level of DT-diaphorase and the inducibility of the enzyme in vitro in tumour biopsy samples prior to the start of therapy.

In summary, this study has demonstrated that inducers of DT-diaphorase can selectively increase the cytotoxicity of MMC in human tumour cells of different tumour type. Enzyme inducers, including dietary components, that produced more induction of DT-diaphorase also produced a greater enhancement of MMC cytotoxic activity. Thus, it may be possible to use non-toxic inducers of DT-diaphorase to enhance the efficacy of bioreductive anti-tumour agents.

### REFERENCES

Adams GE and Stratford IJ (1994) Bioreductive drugs for cancer therapy: the search for tumor specificity. *Int J Radiat Oncol Biol Phys* **29**: 231–238

Appelt LC and Reicks MM (1997) Soy feeding induces phase II enzymes in rat tissues. *Nutr Cancer* **28**: 270–275

Barham HM, Inglis R, Chinje EC and Stratford IJ (1996) Development and validation of a spectrophotometric assay for measuring the activity of NADH:cytochrome b5 reductase in human tumour cells. *Br J Cancer* **74**: 1188–1193

Beall HD, Murphey AM, Siegel D, Hargreaves RH, Butler J and Ross D (1995) Nicotinamide adenine dinucleotide (phosphate): quinone oxidoreductase (DT-diaphorase) as a target for bioreductive antitumour quinones: quinone cytotoxicity and selectivity in human lung and breast cancer cell lines. *Mol Pharmacol* **48**: 499–504

Beall HD, Liu YF, Siegel D, Bolton EM, Gibson NW and Ross D (1996) Role of NAD(P)H:quinone oxidoreductase (DT-diaphorase) in cytotoxicity and induction of DNA damage by streptonigrin. *Biochem Pharmacol* **51**: 645–652

Bedikian AY, Legha SS, Eton O, Buzaid AC, Papadopoulos N, Coates S, Simmons T, Neefe J and von Roemeling R (1997) Phase II trial of tirapazamine combined with cisplatin in chemotherapy of advanced malignant melanoma. *Ann Oncol* **8**: 363–367

Begleiter A, Robotham E, Lacey G and Leith MK (1989) Increased sensitivity of quinone resistant cells to mitomycin C. *Cancer Lett* **45**: 173–176

© 1999 Cancer Research Campaign 

Begleiter A, Robotham E and Leith MK (1992) The role of NAD(P)H:(quinone acceptor) oxidoreductase (DT-diaphorase) in activation of mitomycin C under hypoxia. *Mol Pharmacol* **41**: 677–683
Begleiter A, Verburg L, Ashique A, Lee K, Israels LG, Mowat MRA and Johnston JB (1995) Comparison of antitumor activities of 2-chlorodeoxyadenosine and 9-β-arabinosyl-2-fluoroadenine in chronic lymphocytic leukemia and marrow cells in vitro. *Leukemia* **9**: 1875–1881
Begleiter A, Leith MK and Curphey TJ (1996) Induction of DT-diaphorase by 1,2-dithiole-3-thione and increase of antitumour activity of bioreductive agents. *Br J Cancer* **74**: S9–S14
Belcourt MF, Hodnick WF, Rockwell S and Sartorelli AC (1996) Differential toxicity of mitomycin C and porfiromycin to aerobic and hypoxic Chinese hamster ovary cells overexpressing human NADPH:cytochrome C (P450) reductase. *Proc Natl Acad Sci USA* **93**: 456–460
Belinsky M and Jaiswal AL (1993) NAD(P)H:quinone oxidoreductase 1 (DT-diaphorase) expression in normal and tumor tissues. *Cancer Metastasis Rev* **12**: 103–117
Beyer RE, Segura Aguilar JE and Ernster L (1988) The anticancer enzyme DT diaphorase is induced selectively in liver during ascites hepatoma growth. *Anticancer Res* **8**: 233–238
Boyer MJ (1997) Bioreductive agents: a clinical update. *Oncol Res* **9**: 391–395
Coia LR (1993) The use of mitomycin in esophageal cancer. *Oncology* **50**: 53–62
Cummings BJ, Keane TJ, O'Sullivan B, Wong CS and Catton CN (1993) Mitomycin in anal canal carcinoma. *Oncology* **50**: 63–69
Doherty GP, Leith MK, Wang X, Curphey TJ and Begleiter A (1998) Induction of DT-diaphorase by 1,2-dithiole-3-thiones in human tumour and normal cells and effect on anti-tumour activity of bioreductive agents. *Br J Cancer* **77**: 1241–1252
Egner PA, Kensler TW, Prestera T, Talalay P, Libby AH, Joyner HH and Curphey TJ (1994) Regulation of phase 2 enzyme induction by oltipraz and other dithiolethiones. *Carcinogenesis* **15**: 177–181
Fitzsimmons SA, Workman P, Grever M, Paull K, Camalier R and Lewis AD (1996) Reductase enzyme expression across the National Cancer Institute tumor cell line panel: correlation with sensitivity to mitomycin C and EO9. *J Natl Cancer Inst* **88**: 259–269
Fujita K, Kubota T, Matsuzaki SW, Otani Y, Watanabe M, Teramoto T, Kumai K and Kitajima M (1998) Further evidence for the value of the chemosensitivity test in deciding appropriate chemotherapy for advanced gastric cancer. *Anticancer Res* **18**: 1973–1978
Gustafson DL and Pritsos CA (1992) Bioactivation of mitomycin C by xanthine dehydrogenase from EMT6 mouse mammary carcinoma tumors. *J Natl Cancer Inst* **84**: 1180–1185
Habig WH, Pabst MJ and Jakoby WB (1974) Glutathione S-transferase. The first enzymatic step in mercapturic acid formation. *J Biol Chem* **249**: 7130–7139
Hodnick WF and Sartorelli AC (1993) Reductive activation of mitomycin C by NADH:cytochrome b5 reductase. *Cancer Res* **53**: 4907–4912
Hortobagyi GN (1993) Mitomycin: its evolving role in the treatment of breast cancer. *Oncology* **50**: 1–8
Jaiswal AK (1991) Human NAD(P)H:quinone oxidoreductase (NQO-1) gene structure and induction by dioxin. *Biochemistry* **30**: 10647–10653
Jaiswal AK, Burnett P, Adesnik M and McBride OW (1990) Nucleotide and deduced amino acid sequence of a human cDNA (NQO2) corresponding to a second member of the NAD(P)H:quinone oxidoreductase gene family. Extensive polymorphism at the NQO2 gene locus on chromosome 6. *Biochemistry* **29**: 1899–1906
Johnston JB, Verburg L, Shore T, Williams M, Israels LG and Begleiter A (1994) Combination therapy with nucleoside analogs and alkylating agents. *Leukemia* **8**: S140–S143
Kensler TW and Helzlsouer KJ (1995) Oltipraz: clinical opportunities for cancer chemoprevention. *J Cell Biochem* **Suppl. 22**: 101–107
Kirkpatrick DL, Duke M and Goh TS (1990) Chemosensitivity testing of fresh human leukemia cells using both a dye exclusion assay and a tetrazolium dye (MTT) assay. *Leuk Res* **14**: 459–466
Lown JW, Begleiter A, Johnson D and Morgan AR (1976) Studies related to antitumor antibiotics. Part V. Reaction of mitomycin C with DNA examined by ethidium fluorescence assay. *Can J Biochem* **54**: 110–119
Malkinson AM, Siegel D, Forrest GL, Gazdar AF, Oie HK, Chan DC, Bunn PA, Mabry M, Dykes DJ, Harrison SD and Ross D (1992) Elevated DT-diaphorase activity and messenger RNA content in human non-small cell lung carcinoma: relationship to the response of lung tumor xenografts to mitomycin C. *Cancer Res* **52**: 4752–4757
Manson MM, Ball HW, Barrett MC, Clark HL, Judah DJ, Williamson G and Neal GE (1997) Mechanism of action of dietary chemoprotective agents in rat liver: induction of phase I and II drug metabolizing enzymes and aflatoxin B1 metabolism. *Carcinogenesis* **18**: 1729–1738
Marin A, Lopez de Cerain A, Hamilton E, Lewis AD, Martinez-Penuela JM, Idoate MA and Bello J (1997) DT-diaphorase and cytochrome $b_5$ reductase in human lung and breast tumours. *Br J Cancer* **76**: 923–929
Marshall RS and Rauth AM (1986) Modification of the cytotoxic activity of mitomycin C by oxygen and ascorbic acid in Chinese hamster ovary cells and a repair-deficient mutant. *Cancer Res* **46**: 2709–2713
Mikami K, Naito M, Tomida A, Yamada M, Sirakusa T and Tsuruo T (1996) DT-diaphorase as a critical determinant of sensitivity to mitomycin C in human colon and gastric carcinoma cell lines. *Cancer Res* **56**: 2823–2826
Miller VA, Ng KK, Grant SC, Kindler H, Pizzo B, Heelan RT, von Roemeling R and Kris MG (1997) Phase II study of the combination of the novel bioreductive agent, tirapazamine, with cisplatin in patients with advanced non small cell lung cancer. *Ann Oncol* **8**: 1269–1271
Nishiyama M, Saeki S, Aogi K, Hirabayashi N and Toge T (1993) Relevance of DT-diaphorase activity to mitomycin C (MMC) efficacy on human cancer cells: differences in in vitro and in vivo systems. *Int J Cancer* **53**: 1013–1016
Nishiyama M, Suzuki K, Kumazaki T, Yamamoto W, Toge T, Okamura T and Kurisu K (1997) Molecular targeting of mitomycin C chemotherapy. *Int J Cancer* **72**: 649–656
Plumb JA, Gerritsen M, Milroy R, Thomson P and Workman P (1994) Relative importance of DT-diaphorase and hypoxia in the bioactivation of EO9 by human lung tumor cell lines. *Int J Radiat Oncol Biol Phys* **29**: 295–299
Prestera T, Holtzclaw WD, Zhang Y and Talalay P (1993) Chemical and molecular regulation of enzymes that detoxify carcinogens. *Proc Natl Acad Sci USA* **90**: 2965–2969
Pritsos CA and Sartorelli AC (1986) Generation of reactive oxygen radicals through bioactivation of mitomycin C antibiotics. *Cancer Res* **46**: 3528–3532
Prochaska HJ and Santamaria AB (1988) Direct measurement of NAD(P)H:quinone reductase from cells cultured in microtiter wells: a screening assay for anticarcinogenic enzyme inducers. *Anal Biochem* **169**: 328–336
Riley RJ and Workman P (1992) DT-diaphorase and cancer chemotherapy. *Biochem Pharmacol* **43**: 1657–1669
Rockwell S, Sartorelli AC, Tomasz M and Kennedy KA (1993) Cellular pharmacology of quinone bioreductive alkylating agents. *Cancer Metastasis Rev* **12**: 165–176
Ross D, Siegel D, Beall H, Prakash AS, Mulcahy RT and Gibson NW (1993) DT-diaphorase in activation and detoxification of quinones. *Cancer Metastasis Rev* **12**: 83–101
Ross D, Beall H, Traver RD, Siegel D, Phillips RM and Gibson NW (1994) Bioactivation of quinones by DT-diaphorase, molecular, biochemical, and chemical studies. *Oncol Res* **6**: 493–500
Schellens JHM, Planting AST, Van Acker BAC, Loos WJ, De Boer-Dennert M, Van der Burg MEL, Koier I, Krediet RT, Stoter G and Verweij J (1994) Phase I and pharmacologic study of the novel indoloquinone bioreductive alkylating cytotoxic drug EO9. *J Natl Cancer Inst* **86**: 906–912
Schlager JJ and Powis G (1990) Cytosolic NAD(P)H: (quinone acceptor) oxidoreductase in human normal and tumor tissue: effects of cigarette smoking and alcohol. *Int J Cancer* **45**: 403–409
Smitskamp-Wilms E, Giaccone G, Pinedo HM, Van der Laan BFAM and Peters GJ (1995) DT-diaphorase activity in normal and neoplastic human tissues: an indicator of sensitivity to bioreductive agents? *Br J Cancer* **72**: 917–921
Spain RC (1993) The case for mitomycin in non-small cell lung cancer. *Oncology* **50**: 35–52
Strobel HW and Dignam JD (1978) Purification and properties of NADPH-cytochrome P-450 reductase. *Methods Enzymol* **52**: 89–96
Tomasz M, Lipman R, Chowdary D, Pawlak L, Verdine GL and Nakanishi K (1987) Isolation and structure of a covalent cross-link adduct between mitomycin C and DNA. *Science* **235**: 1204–1208
Wang W and Higuchi CM (1995) Induction of NAD(P)H:quinone reductase by vitamins A, E, and C in Colo205 cancer cells. *Cancer Lett* **98**: 63–69
Winski SL, Hargreaves RHJ, Butler J and Ross D (1998) New aziridinylbenzoquinones as NAD(P)H:quinone oxidoreductase (NQO1)-directed antitumor agents. *Proc Am Assoc Cancer Res* **29**: 303
Workman P and Stratford IJ (1993) The experimental development of bioreductive drugs and their role in cancer therapy. *Cancer Metastasis Rev* **12**: 73–82
Xu BH, Gupta V and Singh SV (1994) Characterization of a human bladder cancer cell line selected for resistance to mitomycin C. *Int J Cancer* **58**: 686–692
Zhang Y, Kensler TW, Cho CG, Posner GH and Talalay P (1994) Anticarcinogenic activities of sulforaphane and structurally related synthetic norbornyl isothiocyanates. *Proc Natl Acad Sci USA* **91**: 3147–3150

 *© 1999 Cancer Research Campaign*
